# Models of Marine Fish Biodiversity: Assessing Predictors from Three Habitat Classification Schemes

**DOI:** 10.1371/journal.pone.0155634

**Published:** 2016-06-22

**Authors:** Katherine L. Yates, Camille Mellin, M. Julian Caley, Ben T. Radford, Jessica J. Meeuwig

**Affiliations:** 1 Australian Institute of Marine Science, PMB 3, Townsville, Queensland, Australia; 2 School of Environment and Life Sciences, University of Salford, Manchester, United Kingdom; 3 Global Change Ecology Lab, School of Biological Sciences, University of Adelaide, Adelaide, South Australia, Australia; 4 Australian Institute of Marine Science, UWA Oceans Institute, Crawley, Western Australia, Australia; 5 Centre for Marine Futures, Oceans Institute and School of Animal Biology, University of Western Australia, Crawley, Western Australia, Australia; Bangor University, UNITED KINGDOM

## Abstract

Prioritising biodiversity conservation requires knowledge of where biodiversity occurs. Such knowledge, however, is often lacking. New technologies for collecting biological and physical data coupled with advances in modelling techniques could help address these gaps and facilitate improved management outcomes. Here we examined the utility of environmental data, obtained using different methods, for developing models of both uni- and multivariate biodiversity metrics. We tested which biodiversity metrics could be predicted best and evaluated the performance of predictor variables generated from three types of habitat data: acoustic multibeam sonar imagery, predicted habitat classification, and direct observer habitat classification. We used boosted regression trees (BRT) to model metrics of fish species richness, abundance and biomass, and multivariate regression trees (MRT) to model biomass and abundance of fish functional groups. We compared model performance using different sets of predictors and estimated the relative influence of individual predictors. Models of total species richness and total abundance performed best; those developed for endemic species performed worst. Abundance models performed substantially better than corresponding biomass models. In general, BRT and MRTs developed using predicted habitat classifications performed less well than those using multibeam data. The most influential individual predictor was the abiotic categorical variable from direct observer habitat classification and models that incorporated predictors from direct observer habitat classification consistently outperformed those that did not. Our results show that while remotely sensed data can offer considerable utility for predictive modelling, the addition of direct observer habitat classification data can substantially improve model performance. Thus it appears that there are aspects of marine habitats that are important for modelling metrics of fish biodiversity that are not fully captured by remotely sensed data. As such, the use of remotely sensed data to model biodiversity represents a compromise between model performance and data availability.

## Introduction

Globally, marine biodiversity provides myriad and valuable ecosystem goods and services [1–3]. These goods and services, however, are threatened by an extensive range of natural and anthropogenic stressors, many of which are broadly distributed [4,5]. Consequently, many of these goods and services are in decline [2]. The conservation and sustainable management of marine biodiversity is hampered by insufficient information [5] and taxonomic bias in the information currently available [6]. Effective conservation and management is further impeded by the difficulty and cost of acquiring additional data from marine habitats due to their extent and inaccessibility, as well as the complexity of these systems. The lack of effective biological surrogates [7,8], and the often insufficient opportunities to supplement current information, means that other approaches must be used to overcome these information and knowledge gaps.

Recent advances in collecting and analysing marine data are supporting new analytical techniques that can help fill these gaps, and in doing so, contribute to more effective conservation and management of living marine resources [9,10]. Tools such as baited cameras and towed video enable direct observation of marine species and their habitats, in more affordable and efficient ways, and in places divers cannot access [11–13]. Multibeam sonar is also now commonly used to generate high-resolution bathymetry of marine habitats (e.g. [10]). In turn, such bathymetry supports habitat classification at very fine spatial resolution over large areas of seafloor [10,14,15].

More commonly now, predictive biodiversity models developed with remotely sensed data are being developed and applied to marine ecosystems. However, whether predicted habitat classification provides better predictors for modelling metrics of biodiversity (e.g. species richness) than predictors taken directly from remotely sensed data is not yet known. Similarly, predictors from remotely sensed data have not previously been compared to predictors obtained from direct observer habitat classification. Assessment of the strength of predictors produced by these three methods could improve our understanding of how to build better models by informing the selection of sampling methods, and the suitability of such models according to their intended use and the relative resource requirements of information acquisition. Furthermore, the use of such models to predict assemblage-level metrics has, so far, been largely limited to predicting total species richness, total abundance or total biomass (e.g. [16]). Utility of these models would be enhanced if they could predict a wider suite of metrics that are informative with respect to conservation and management objectives. Such metrics might include abundance and biomass of specific groups such as vulnerable species or endemic species, or functional group membership more broadly.

Here we investigate which of a range of habitat classification methods provides the most powerful predictors when modelling metrics of fish biodiversity and explore which of a series of metrics can be most effectively modelled. We use data from the Marine Futures Project (2005–2008), funded through Australia’s Natural Heritage Trust. Using these data, we test the predictive power of environmental parameters obtained in three different ways: acoustic (multibeam) sonar imagery, predicted habitat classification, and direct observer habitat classification. We then investigate the relative influence of individual predictors and test the ability of models to predict a series of community-level univariate and multivariate metrics: total species richness, total abundance, total biomass, abundance and biomass of vulnerable species (‘vulnerability’, after [17]), abundance and biomass of fisheries targeted species, abundance and biomass of endemic species, and functional group composition by abundance and biomass.

## Methods

### Study Site

**Fig 1:**
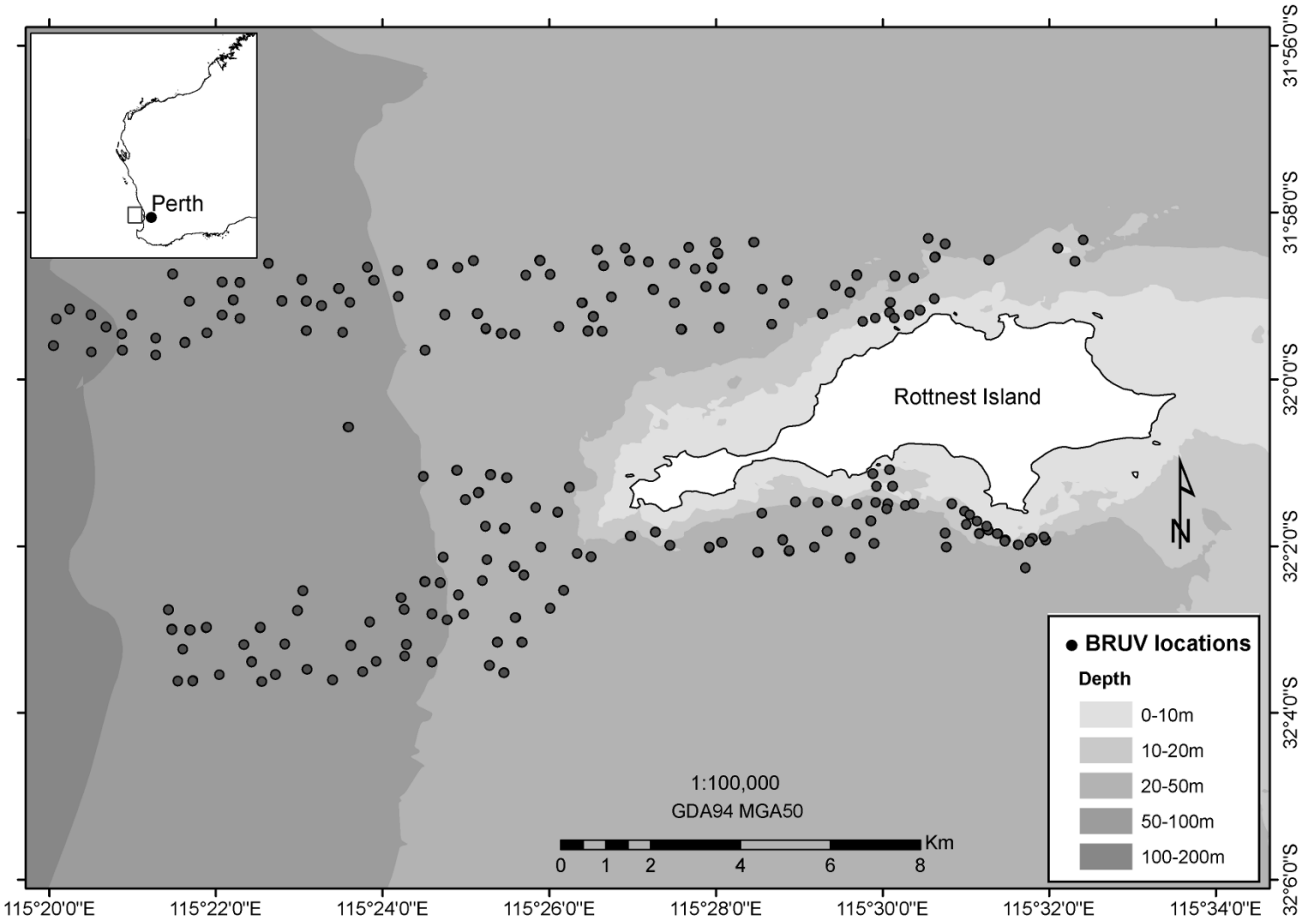
Map of Rottnest study site, Western Australia. Geographic Datum of Australia 94 Zone 50 South (GDA 94 Z50).

Rottnest Island (Fig 1, Geographic Datum of Australia 94 Zone 50 South, GDA 94 Z50) is biologically diverse, and includes a wide range of habitats from tropical coral reefs to rocky temperate reefs, seagrass beds and sandy barrens. This diversity reflects the strong influence of the Leeuwin Current, a south-flowing boundary current that brings warm tropical water and species into this otherwise sub-tropical region. As such, the region supports an unusual mix of tropical and temperate fishes [18]. Rottnest Island and its environs are also an important recreational area in close proximity to Western Australia’s state capital, Perth, and as such, marine based tourism and recreational activities including sailing, diving and fishing are highly valued [19]. There is limited commercial fishing in the area and no oil and gas exploration activity. Despite the area’s high conservation and socioeconomic value, there is little information on the distribution of its marine biodiversity, and therefore, the implication of these distributions for management.

Rottnest Island was surveyed in 2007 as part of the Marine Futures Program (www.marinefutures.fnas.uwa.edu.au). Surveys included multibeam mapping, towed video and 349 Baited Remote Underwater Video (BRUV) deployments. From these surveys the species data (fish) and habitat data (three types) used in this study were obtained. These data and the methods used to obtain them are described below.

### Habitat data

An area around Rottnest (~250 km^2^) was subject to a full coverage multibeam survey conducted by Fugro (PTY). The multibeam survey was conducted using a Reson 8101 Multibeam. The multibeam sensor point cloud varied with water depth but on average collected one point or ping every ~50-100cm, which was averaged over a 2.5m cell. Development of secondary datasets from the hydroacoustic data provided textural information about the seafloor. Data were processed to construct full coverage bathymetry maps with a 2.5m cell size. Combined, these data formed our first group of (27) habitat variables, hereafter referred to as multibeam habitats.

Approximately 100 km of towed video imagery was also collected. Processing of this imagery consisted of classifying the observed imagery with respect to habitat type and geo-referencing position such that boundaries between habitat types, and thus patches of habitat types, were identified ([20]). Habitat types were classified by trained and cross-validated analysts into 34 broad classes using a standard classification scheme for Australia [21]. The multibeam and the habitat classification data from the towed video were used to generate predicted habitat classifications across the study area, again at a cell size of 2.5m. Regression trees [22,23] were used to predict the probability of the presence of a given habitat type in a given cell (See S1 File, supplementary methods, for full details). These data formed our second group of (34) habitat variables, hereafter referred to as predicted habitats.

Lastly, during the analysis of the BRUVS images, habitats observed were classified by visual observation in to one of five abiotic habitat classes (high, medium and low profile reef, sand inundated reef, or sand) and one of six biotic habitat classes (macroalgae, seagrass, coral, sessile invertebrates, bare). These data formed our third group of (2) categorical habitat variables (abiotic and biotic categories), hereafter referred to as direct observer habitats.

### Species Data

The predicted habitat map generated from the multibeam and towed video formed the basis of the sampling design for the fish survey using stereo-BRUVS. A total of 349 BRUV deployments (hereafter referred to as samples) were made at the Rottnest site. All samples with visibility ≤ 3m were excluded, leaving a total of 280 samples used in this study. From each of the remaining samples, fish species were identified and there relative abundances and individual fork lengths were estimated using Event-Measure [24]. See S1 File, supplementary methods, for full details of BRUVS sampling design and image analysis.

A series of biodiversity metrics were calculated for each sample (Table 1). These metrics were derived from species identifications, relative species abundances and individual fork lengths. Biomass was estimated by calculating individual fish weight from fork length using relationships available on FishBase [25]. Fishbase was also used: to assign each species to a trophic level and score their vulnerability to exploitation [17], to class each species as either endemic to Australia or not, to assign each species to a functional group, and to ascertain whether or not each species was targeted by fisheries [25].

**Table 1:** Estimates of biodiversity metrics for marine fishes around Rottnest Island, Western Australia. Vulnerability, target and endemic metrics are reported as a percentage of the total abundance or total biomass. Results are from 280 samples, each sample being an individual BRUVs deployment.

Metric	Mean	Std.Dev	min	max
Total species richness	10.8	5.8	1	31
Total abundance	55.5	75.2	1	1041
Total biomass (g)	33252.1	51675.2	67.9	735990.2
Vulnerability (abundance)	4.3	8.9	16.9	87
Vulnerability (biomass)	61.8	11.4	24.2	87
Target (abundance)	54.1	26.8	0	100
Target (biomass)	77.6	23.1	0	100
Endemic (abundance)	83.1	21.7	0	100
Endemic (biomass)	48	31.9	0	100

Vulnerability estimates were based on species’ life history traits and scored on a scale of 1 (lowest) to 100 (highest) [17]. Classification to functional group was based on information on prey items, trophic level and maximum body size, as reported in Fishbase (see S1 File, supplementary methods).

The large number of benthic omnivores and zoobenthivores and the large variations in their body sizes led us to separate them into small and large size classes within both groups of species. Small and large omnibenthivores were separated at a mean observed size of 31.5 cm while small and large zoobenthivores were separated at a mean observed size of 35.0 cm. The classification of individuals into small and large categories for the omnibenthivores and zoobenthivores was based on the distribution of observed sizes, with the former trophic category tending towards smaller individuals.

### Modelling

#### Variable selection

Our set of potential predictor variables was large and included estimates of 27 multibeam derived variables and 34 predicted habitat variables, plus two direct observer habitat variables (abiotic & biotic categories) and depth (see S1 Table for a detailed description). Whilst regression trees largely ignore non-informative predictors when fitting trees [26,27], predictor selection is still useful because redundant predictors can degrade a model’s performance by increasing variance (Elith *et al* 2008). BRT are also only robust to moderate levels of multicollinearity [28]. Therefore, within each of the multibeam and predicted habitat variable sets, we selected predictors for use in our models based on three steps. We identified predictors that were highly correlated (Pearson’s *r* > 0.8). We developed an initial set of exploratory BRT models including all candidate predictors and examined their relative influence, identifying candidate predictors for which the relative influence was consistently ≤ 1%. Finally, we ran a step-wise simplification of those exploratory models, using methods analogous to backward selection in regression (after Elith *et al* 2008), to see which variables were repeatedly dropped from the models. Based on our initial investigation of these candidate predictors, we reduced our predictors to 12 multibeam variables, 11 predicted habitat variables, the two direct observer habitat variables (abiotic and biotic categories) and depth (S4 Table).

#### Boosted regression trees

We used boosted regression trees (BRT) (Elith *et al*. 2006, 2008) to model relationships between environmental characteristics and nine univariate, community level metrics (Table 1). Models were developed in R 3.1.1 [29], using the gbm package.

Distributions of community metrics were skewed. Therefore, we either log(x+1) transformed them if variables were null or positive (species richness, total abundance and total biomass) or logit transformed them if bounded between 0 and 1 (vulnerability, target and endemic metrics) to approximate a normal distribution [26,30]. For each community metric, models were developed for five sets of predictors: 1) multibeam plus direct observer habitats, 2) predicted habitats plus direct observer habitats, 3) multibeam, 4) predicted habitats, 5) direct observer habitats. All models also included depth.

**Fig 2:**
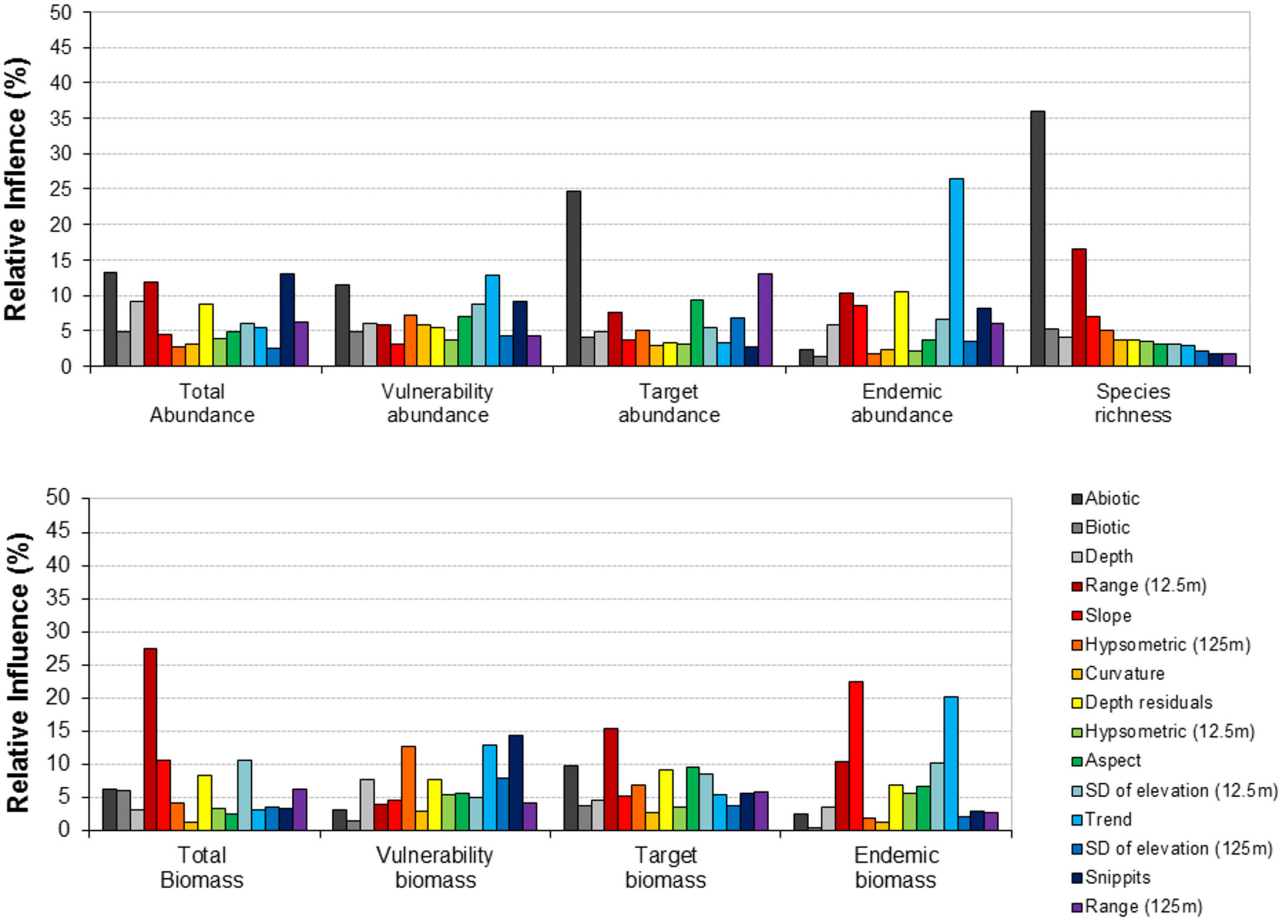
The relative influence (%) of individual predictors estimated using boosted regression tree models for nine fish diversity metrics. Models were developed with predictors from Multibeam data plus direct observer habitats (abiotic and biotic) and depth. Multibeam data and depth were numeric values, the abiotic and biotic variables were categorical (five abiotic and six biotic classes). Vulnerability, target and endemic metrics were developed for both the percentage of the total abundance and the percentage of the total biomass.

**Fig 3:**
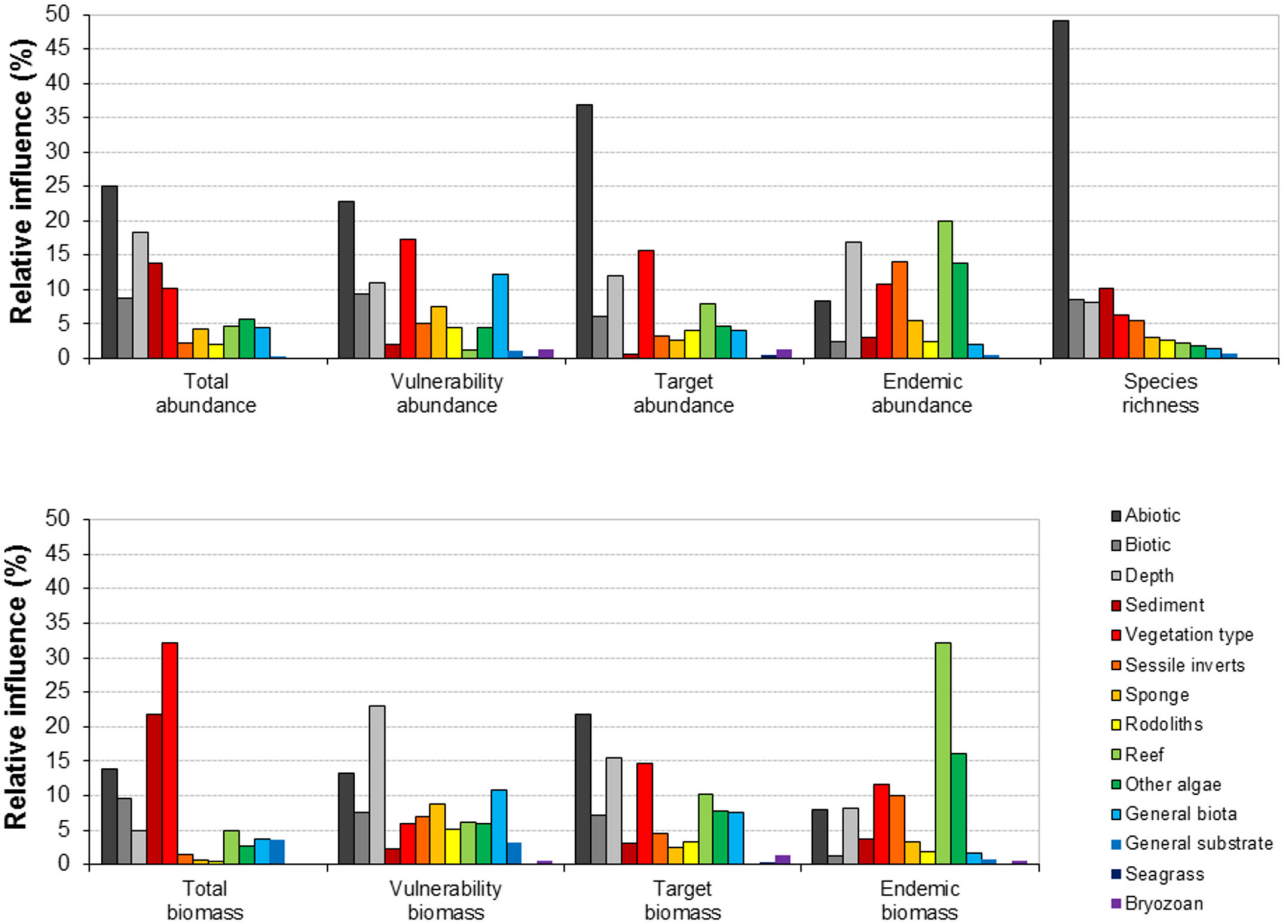
The relative influence (%) of individual predictors as estimated using boosted regression tree models for nine fish diversity metrics. Models were developed with predictors from predicted habitat classes, plus direct observer habitats (abiotic and biotic) and depth. Predicted habitat variables are numeric and describe the proportion of that habitat within the sample area, the abiotic and biotic variables were categorical (five abiotic and six biotic classes). Vulnerability, target and endemic metrics were developed for both the percentage of total abundance and the percentage of the total biomass.

Models were developed using a randomly selected 50% of the data (0.5 bag fraction). Following Elith et al. (2008), the 50% level was chosen based on model performance after a range of values between 30 and 70% were tested on an initial set of models, and their predictive powers compared. Cross-validation (CV), using bootstrapped data, was used to evaluate the predictive capacity of the models. For each combination of community level metric and set of predictors, a range of values for the two main model parameters, tree complexity and learning rate, were explored and the best model selected based on the greatest CV deviance explained and lowest mean prediction error, following [26] (see S2 Table for the parameters of the best models). Model residuals were tested (Moran’s I) to rule out spatial autocorrelation. In some cases, intra-model variability was of similar magnitude to inter-model variability, so each model was run five times with the same parameters and the average CV deviance and prediction error were calculated across model runs (S3 Table shows the ranges in CV deviance and prediction error for the best models). We also examined the relative influence (%) of each predictor on the best models (Figs 2 and 3).

#### Multivariate regression trees

We used multivariate regression trees (MRT) (De'Ath 2002) to model the relationship between predictors and the relative abundance (or biomass) of the different trophic and vulnerability-based functional groups (R package mvpart). We defined groups by combining diet and vulnerability, which resulted in 29 functional groups ranging from ‘high vulnerability piscivores’ to ‘low vulnerability zooplanktivores. We built an initial model using predictors from the multibeam data, which we then compared to a second model built using predicted habitat classes derived from the multibeam—both models also included the direct observer habitats (biotic and abiotic categories) and depth.

MRT clusters sites by repeated splitting of the data, with each split determined by habitat characteristics [31] and corresponding to a distinct species assemblage. Tree fit is defined by the relative error (RE; total impurity of the final tree divided by the impurity of the original data). RE is an over-optimistic estimate of tree accuracy, which is better estimated from the cross-validated relative error (CVRE). We determined the best tree size (i.e. number of leaves or clusters formed by the tree) as that which minimized CVRE, which varies from zero for a perfect predictor to nearly one for a poor predictor (De'Ath 2002). We then examined the splits and quantified the variance that each of them explained, based on the entire dataset and for each individual functional group.

We identified functional groups and species that characterized each resulting cluster using the Dufrêne-Legendre index, which is based on the relative abundance and frequency of each species (or functional group) within a given cluster [32]. The index varies between 0, no occurrences of a species within a cluster, to 100, if a species occurs at all sites within the cluster and in no other cluster. The index is associated with the probability of resulting from a random pattern, based on 250 reallocations of sites among clusters[32].

### Ethical approval

Quantitative measurements of the fish assemblage were made using non-destructive sampling methods (BRUVS) and there was no sacrifice or incident mortality associated with this sampling method. The use of BRUVS for collecting the data used here required ethics approval, which was from the University of Western Australia and approval from the Western Australian Department of Fisheries. No other approvals were necessary, nor did sampling occur within protected areas or on private property. The field work did not impact any listed protected species.

## Results

### Boosted regression trees

BRT explained up to 63.9% CV deviance in response variables, depending on the set of predictors used and the community metric being modelled (Table 2). The best performing models were for total species richness (63.9% CV deviance explained) and total abundance (43.4% CV deviance explained). Biomass proved more difficult to model, with CV deviance explained consistently lower for biomass than for the corresponding abundance (Table 2). Abundance and biomass of endemic species proved the most difficult to model, with a CV deviance explained of only 10.0% and 8.4%, respectively.

**Table 2:** Results of boosted regression tree models for nine biodiversity metrics, developed with five sets of predictors. Predictors were developed from three sets of habitat data: multibeam habitats (multibeam), predicted habitats (pred. habitats) and direct observer habitats (biotic and abiotic categories). All models also contained depth as a predictor. Vulnerability, target and endemic metrics were developed for both the percentage of the total abundance and the percentage of the total biomass. The cross validation (CV) deviance explained (%) and mean prediction error (%) are averaged values from five model runs. The greatest CV deviance explained and the lowest prediction error for each metric are highlighted in bold. Data were collected around Rottnest Island, Western Australia, in 2007.

		Predictors				
	Metrics	Multibeam, depth, biotic & abiotic	Pred. habitats, depth, biotic & abiotic	Multibeam & depth	Pred. habitats & depth	Biotic, abiotic & depth
**CV deviance explained (%)**	Total species richness	**63.9**	63.3	55.8	49.9	58.5
	Total abundance	**43.4**	42.1	38.9	34.6	39.6
	Total biomass	16.6	**17.6**	14.8	16.1	14.1
	Vulnerability (abundance)	25.9	**27**	22.1	23.5	23.4
	Vulnerability (biomass)	0.5	**1.9**	0.4	1.2	1
	Target (abundance)	**26.9**	**26.9**	19	11.8	24.3
	Target (biomass)	**12.1**	9	8.5	3	7
	Endemic (abundance)	8.5	**10**	8.2	9.3	5.5
	Endemic (biomass)	6.3	**8.4**	6.2	8.3	2.7
**Mean prediction error (%)**	Total species richness	**10.4**	11.9	11.5	14.6	13.4
	Total abundance	**13.4**	15.4	17	19.8	18.2
	Total biomass	**8.1**	8.5	8.3	8.6	8.7
	Vulnerability (abundance)	**10.3**	11.9	11.2	12.3	12.2
	Vulnerability (biomass)	**14.9**	15.4	15.5	15.6	15.8
	Target (abundance)	**64.5**	77.2	71	94.6	76.5
	Target (biomass)	**69.7**	70	85.7	97.9	85
	Endemic (abundance)	**48.9**	69.3	52	64.6	55
	Endemic (biomass)	**234.8**	245.3	250.3	252.7	313.4

Models developed using predictors from predicted habitats, plus direct observer habitats and depth generally had the greatest CV deviance explained, outperforming models developed with other sets of predictors for five out of nine metrics (Table 2). Models that excluded the direct observer habitats consistently performed worse than corresponding models including them, highlighting the importance of these two variables. Indeed, models including depth and the direct observer habitats alone were able to explain >80% of the maximum CV deviance for five of the nine community metrics, and >50% for eight of the nine metrics (across all metrics, range = 35–95%, mean = 70%).

Models without direct observer habitats also performed worse in terms of mean prediction error than corresponding models that included them (Table 2). In almost all cases, models for biomass had greater mean prediction error than the corresponding models for abundance, again indicating that biomass was less predictable than abundance. Models developed using multibeam data had consistently lower prediction error than those developed with predicted habitats, however, the difference was generally very small (i.e. ≤ 0.01 for five of the nine metrics; Table 2).

When using either multibeam or predicted habitats, plus direct observer habitats and depth, the abiotic variable from the direct observer habitats generally had the greatest relative influence on BRTs, with 22.1% average influence on models developed using habitats and 12.2% on models developed using multibeam (Figs 2 and 3). Relative influence was much more evenly distributed between individual predictors for models developed using multibeam habitats than those developed from predicted habitats (Figs 2 and 3). The relative influence of individual predictors varied greatly across community metrics; the abiotic category for example had a much greater influence on abundance metrics (28.5% ± 13.4 when using habitats and 17.6% ± 11.5 when using multibeam; mean ± SD) than it did on biomass metrics (14.2% ± 13.6 when using habitats and 5.3% ± 11.3 when using multibeam).

### Multivariate regression trees

**Fig 4:**
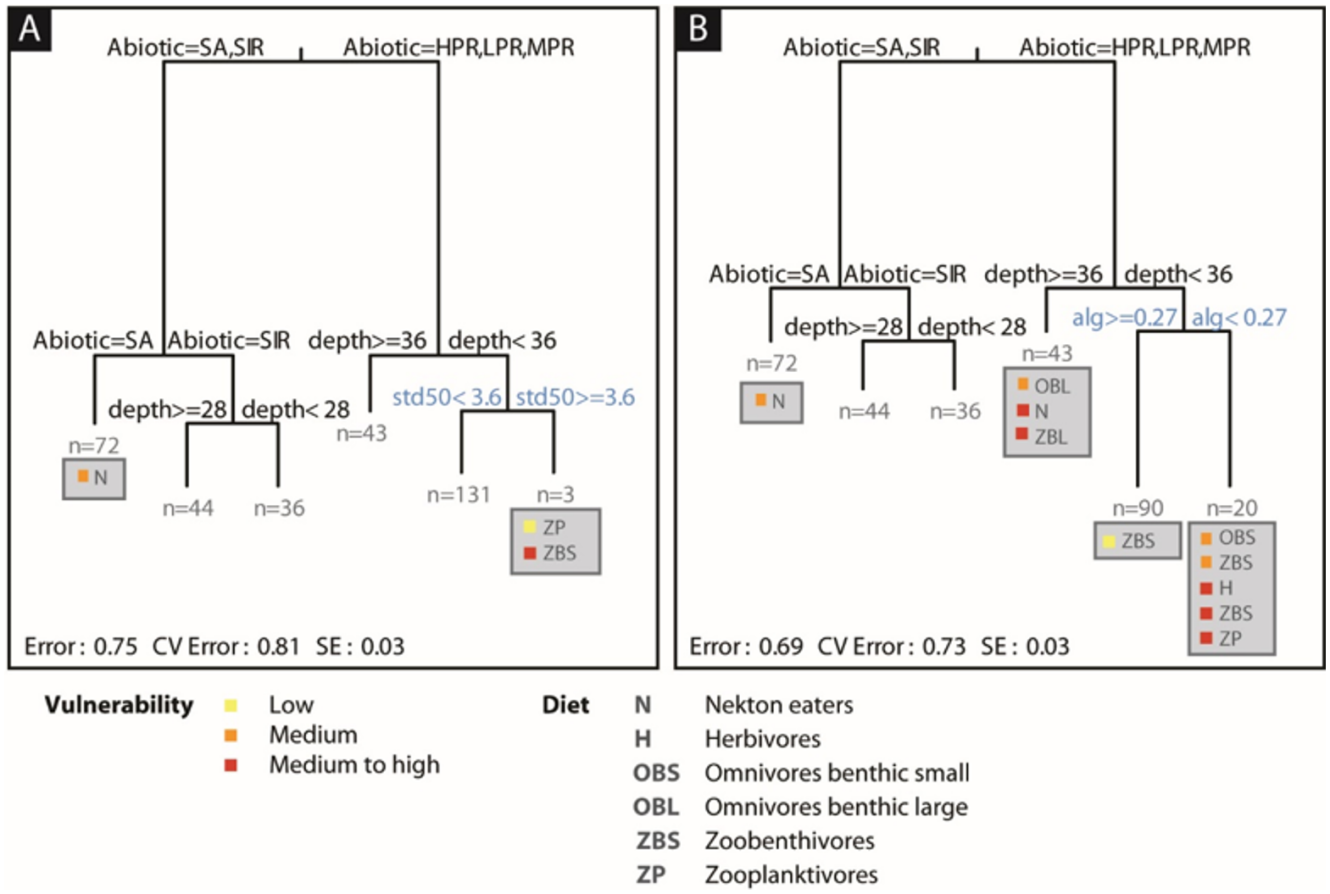
Multivariate regression tress (MRT) of fish functional groups based on A) multibeam data and B) predicted habitats data. Both MRTs also contained depth plus abiotic and biotic predictors from direct observer habitat classification. Multibeam and predicted habitat data were numeric; the abiotic and biotic variables were categorical (five abiotic and six biotic classes). SA = sand, SIR = sand inundated reef, HPR/MPR/LPR = high/medium/low profile reef.

Multivariate regression trees based on either the multibeam or predicted habitats, with the direct observer habitats and depth, were similar, explaining 25% and 31% of variance in assemblage composition, respectively (Fig 4). In both cases, the abiotic variable from the direct observer habitats was associated with the first split, explaining 38% of the variance in assemblage composition by differentiating sandy (16%) from reef habitats (22%). Depth further differentiated shallow (3%) from deeper habitats (3%). Only the last split differed between models, based on either the standard deviation in elevation within a 50-km radius from the multibeam variables (Fig 4A) or algal diversity from predicted habitats (Fig 4B). This last split had little relevance in the first (multibeam) case, only differentiating three samples characterized by a higher abundance of zooplanktivores (*Chromis westaustralis*). By contrast, in the second (predicted habitats) case, the last split led to a more even partitioning of samples, with the last cluster being characterized by medium to high vulnerability omnivores (*Parma mccullochi*), herbivores (*Meuschenia hippocrepis*), zooplanktivores (*Pseudocaranx* sp.) and zoobenthivores (*Coris auricularis*, *Notolabrus parilus*, *Pseudolabrus biserialis*, *Ophtalmolepis lineolata*, *Epinephelides armatus*, *Thalassoma lunare*).

The same models without the direct observer habitats explained only 3% of variance in the first case (i.e. multibeam; CVRE = 1.01), but 24% in the second case (i.e. predicted habitats; CVRE = 0.98). The latter tree consisted of a single split (general biota, *gen_bio*). Multivariate regression trees of fish biomass performed poorly with only 12–14% variance explained depending on the predictors used (RE 0.86–0.88; CVRE 0.88–1.01).

## Discussion

Better predictive models of biodiversity are needed to support conservation and management of marine ecosystems under increasing anthropogenic pressures. Here we explored the use of habitat data obtained by three different classification methods for the development of models predicting a range of both uni- and multivariate fish biodiversity metrics. While in most cases, reasonable models could be developed with remotely sensed data, models that incorporated direct observer habitats consistently outperformed those that did not. Models for abundance also consistently outperformed corresponding models for biomass, and metrics of endemism were particularly difficult to model.

Remote sensing methods, such as multibeam, can provide valuable data for predicting biodiversity metrics [9,10]. Here, models developed using multibeam habitats alone were able to explain a substantial proportion of CV deviance for fish total species richness (56%) and total abundance (39%). Other studies predicting similar metrics from remotely sensed data have obtained similar or even better performance [16,33,34]. As such, our results reaffirm that predictors generated by remote sensing can offer substantial utility in conservation planning and resource management. However, our results also illustrate limitations in the ability of remotely sensed data to predict biodiversity metrics.

Whilst remotely sensed data have been used to model fish species richness, abundance, and biomass (e.g.,[33]), such models have not previously been compared to models generated using alternative predictors. Here, when we did so, models generated with multibeam habitats alone were consistently outperformed by those that included direct observer habitats. The abiotic variable from the direct observer habitats was also the single most influential predictor, possibly because multibeam data does not always enable differentiation between the presence or absence of certain habitats. For example, no detectable difference in multibeam imagery was found before and after the experimental removal of 100m^2^ of kelp from three separate sites [15]. Studies have also shown that seagrass beds can be difficult to detect based on multibeam imagery, particularly when plants are present at low densities [35,36] or when plants are associated with certain substrate types [37]. Thus, there may be ecologically important aspects of the environment that are not captured well in multibeam data, but that are captured by direct observer habitat classification.

Consequently, modelling biodiversity with remotely sensed data represents a compromise between model performance and data availability. While predictors generated from direct observer habitat classification may provide valuable additional information when modelling fish biodiversity metrics, they can be prohibitively expensive to gather at sufficient resolution over large areas like Western Australia. Indeed, estimating biodiversity features over relatively large scales is a key problem [16,38]. Over these large scales remote sensing may offer the only cost-feasible way of generating high-resolution, continuous spatial data that can be used to generate predictions of biodiversity features, such as habitat classes or community level metrics.

Producing predicted habitats requires an additional resource investment, beyond the collection of remotely sensed data. Here, this greater investment did not necessarily result in better predictors for modelling fish biodiversity metrics. For five of the univariate metrics, models developed using predicted habitats resulted in higher CV deviance explained than those developed using multibeam habitats. However, for the other 4 univariate metrics we observed the opposite and in both cases the difference in CV deviance explained was small. BRT models built using multibeam habitats also had consistently lower mean prediction error than those developed with predicted habitats, which suggests that when predicting univariate metrics such as species richness over broad spatial scales, predictors from multibeam data may be more robust. Using multibeam data directly also avoids issues involved in the development of habitat classification models, such as the lack of a standardised method of classification, variable classification accuracy and variable amounts of ground-truthing [10]. Habitat classification maps based on remote sensing are an important part of many marine spatial conservation planning processes [39–42]. However, if habitat maps are not already available for a particular area of interest, our results suggest there may be little to no value in investing additional resources to develop them for the purpose of providing predictors for modelling univariate metrics of biodiversity. There is some indication here that predicted habitat classifications may provide better predictors when developing multivariate models, though more research is needed to understand this potential.

Some biodiversity metrics were more difficult to predict than others. Here, biomass was more difficult to predict than abundance, with lower CV deviance explained and higher mean prediction error. Because biomass is a function of abundance and individual length, estimates of biomass are likely to be more uncertain than non-composite metrics because of compounding errors [43]. Whilst all of the metrics explored here could provide valuable information for conservation planning, our results suggest that some, such as those for endemic species, may not be tractable with predictors from habitat data alone. Modelling others, such as abundance of fisheries targeted species, may require more habitat data than can be generated from remote sensing or predicted habitats alone, and thus, would have limited applicability. Nonetheless, we were able to generate reasonable predictive models of the abundance of vulnerable fishes using only multibeam or predicted habitat data. Having a metric of fish vulnerability provides an important planning tool for both conservationists and fisheries managers [17]. Modelling fish vulnerability over areas such as Western Australia would provide valuable data on where to prioritise effort for both maintaining fish biodiversity and fish stocks, and could inform the positioning of spatial management units such as protected areas or localised fishing-gear modifications.

Biodiversity metrics are impacted by factors other than environmental conditions, and fish metrics will very likely be impacted by fishing. Through the selective extraction of targeted species, fishing alters the composition of communities such that the remaining fishes do not fully reflect the original community composition that would otherwise be determined by local biotic and abiotic conditions. Fishing impacts are very likely to have been present at Rottnest Island as it is a popular location for boat-based recreational fishing [44] and key target species are classified as overexploited in this area [45]. Because fishing generally targets larger individuals of higher trophic level species [46,47], fishing could be expected to have a greater impact on measures of fish biomass than abundance, which may be an additional cause of the poorer performance of the biomass models observed here. Indeed, incorporating fishing pressure, or spatial closures to fishing, alongside environmental predictors can improve model performance when predicting community assemblages [48] and classifying benthic biotopes [49]. Therefore, exploration of ways to incorporate fishing into predictive models of fish biodiversity metrics, over broad spatial scales, would likely be valuable. However, data on the spatial distribution of fishing effort often does not exist [50] or is not accessible [51]. Thus, future studies exploring the incorporation of historical and/or current fishing pressure may need to investigate the use of surrogates for fishing.

Individual habitat predictors had varying influence on the models of fish biodiversity metrics tested here, reflecting how the different metrics respond to different environmental drivers. Fish species richness can be influenced by habitat complexity (e.g. [52]), with increasing complexity leading to increased richness. In general sand patches are species poor, reefs are species rich, and significantly more species are found on high profile rather than low profile reefs [53,54]. Here we found that species richness was best predicted by the abiotic variable from the direct observer habitats, which consisted of five classes that relate very closely to habitat complexity: high/medium/low profile reef, sand inundated reef, sand. Furthermore, the second most influential predictor for species richness was ‘range’, a multibeam variable that describes the topographic variation in an area. The abiotic variable from the direct observer habitats and/or the variable range from the multibeam habitats were also the most influential predictors for the abundances and biomasses of fisheries targeted species, possibly reflecting the association of many of these species with higher complexity reefs. For endemic species (abundance and biomass) the relative influence of either the abiotic variable from the direct observer habitats or range from multibeam habitats was minimal. However, the predicted habitat class ‘reef’ (probability of occurrence) was the most influential of the non-multibeam predictors. This suggests that whilst many endemic species may be reef associated, they are not, as a group, associated with a particular level of reef profile/habitat complexity. Improved understanding of the relative influence of environmental drivers of a broader set of biodiversity metrics, such as those modelled here, could contribute to improved spatial conservation planning processes.

BRT and MRT are emerging techniques for modelling nonlinear species-habitat relationships. Both methods can accommodate different predictor types. Here, for example, we used both categorical and continuous data. These methods can also accommodate missing data, fit complex nonlinear relationships and model interaction effects between predictors [26,27]. When modelling fish species richness, BRT has outperformed more established methods such as multiple linear regression, general adaptive models and neural networks on multiple occasions [16,38,55,56]. Both BRT and MRT can be used to predict across sites for which only environmental data are available [26,27]. Thus BRTs and MRTs could be used to generate maps of predicted biodiversity metrics from remotely sensed data over large areas, which could complement predicted habitat maps and aid conservation planning and natural resource management. Here we demonstrate that at least three community level metrics (total species richness, total abundance and abundance of vulnerable species) can be usefully predicted using BRT and remotely sensed data alone, and as such, how BRT models could inform conservation planning and natural resource management over large, data limited areas, such as the coastal seas off Western Australia. Ultimately, the utility of using remotely sensed data in BRT and MRT models to generate predictions of biodiversity metrics over large areas will depend on the transferability of models generated with sample data collected over a limited number of sites to un-sampled areas [57]. Future studies now need to assess how transferable such models are, and investigate, the factors that impact model transferability.

## Conclusion

Improved understanding of the distribution of a wide range of biodiversity metrics would have great utility for conservation and management, especially if that understanding was based on remotely sensed data. However, it seems that less standard metrics of biodiversity, such as vulnerability to exploitation or endemism, will be difficult to model. Moreover, whilst remotely sensed data can adequately predict total species richness, total abundance, and total biomass, predictive performance of such models can be improved by the addition of data from direct observer habitat classification. As such, it appears that the drivers of some metrics of fish biodiversity are not captured by habitat classification, and that there are aspects of habitats that are important for predicting fish biodiversity that are not readily captured through the use of remotely sensed data alone.

## Supporting Information

S1 File Supplementary materials: additional methods and results. (DOCX)

S1 Table Descriptions of all potential predictor variables. (DOCX)

S2 Table Parameters for ‘best’ BRT models. (DOCX)

S3 Table Ranges of CV Deviance and MPE for ‘best’ BRT models. (DOCX)

S4 Table Selected predictor variables. (DOCX)
