# Supplementary material for: Models of Marine Fish Biodiversity: Assessing Predictors from Three Habitat Classification Schemes
Source: PLoS One. 2016 Jun 22;11(6):e0155634. doi: 10.1371/journal.pone.0155634 (PMC4917103; doi:10.1371/journal.pone.0155634)
Supplement: S1 File — (DOCX) [file pone.0155634.s001.docx]

**Supplementary Materials**

**Methods**

**Sampling design for towed video**

We used a spatially stratified random approach, called Generalised Random Tessellation Stratified (GRTS), to select video transects [1]. The stratification was based on a combination of depth, slope, aspect and rugosity variables obtained from the multibeam data, which were inputs into an isoclass cluster analysis. Stratification was achieved by combining all standardized mutlibeam rasters using a principal components analysis and then using a unsurprised cluster analysis to extract homogeneous groups ( equivalent to geomorphic classes) from Principal Component 1 and 2 which made up 94 % of explained variance [2] . The GRTS transects were then allocated to the different clusters. This ensured both that the spatial extent of each study area was covered, as well as the different (multibeam) habitats. Random allocation was used to ensure unbiased sampling, and distance controls were used to avoid spatial autocorrelation. This provided a habitat-stratified, spatially weighted sampling design covering the study area.

**Sampling design for the fish survey using stereo-BRUVS**

Sampling was stratified by depth and habitat with adjacent sites separated by at least 250–400 m to reduce the likelihood of fish moving between sites [3]. Stereo-BRUVS were deployed following standard methods [4–7]. Stereo BRUVs were deployed by boat and left to film for 60 min on the seafloor. Multiple stereo BRUVs were deployed concurrently at different sites to maximise sampling efficiency. Bait consisted of 800 g of pilchards (*Sardinops* spp.) in a plastic-coated wire mesh basket that was suspended 1.2 m in front of the two SONY handycams (models used included HC 15E, CX7, CX12) mounted 0.7 m apart on a base bar and inwardly converged to provide an overlapping field of view from approximately 0.5 m in from of the cameras.

**Visual habitat classification for towed video**

Biological habitat and abiotic substrate groups were classified by trained and cross-validated visual observation using the CATAMI classification scheme (see <http://catami.org/classification> and [8]). The scheme is designed specifically for Australian waters and allows underwater images to be classified using the same consistent identifiers. Photographs and descriptions of each category are provided in the classification manual. The classification is hierarchical, with scoring to the lowest level distinguishable or needed. In many branches of the classification system classification is taxonomic and can go to species level, however here we used relatively broad, readily identifiable classes (see list below).

| **Towed video images** |
| --- |
| Algae - mixed |
| Algae - red |
| Algae -understory |
| Ascidians |
| Brown algae |
| Bryozoans |
| Coral |
| General Substrate |
| Gorgonians |
| Gravel |
| Hard coral |
| High profile reef |
| Kelp |
| low profile reef  Macroalgae |
| Medium profile reef |
| No biota |
| Non coral sessile inverts |
| Other algae  Rhodoliths |
| Sand |
| Sand inundated reef |
| Scytophalia |
| Seagrass |
| Seagrass - amphibolis |
| Seagrass - posidonia |
| Sediment |
| Sediment - fine |
| Sediment medium |
| Sediment - coarse |
| Sessile Invertebrates |
| Sponge |
| Vegetation |

**Predicted habitat generation**

The relationships between the video image classification and the multibeam depth and derivatives were characterised using regression trees ([9,10] using the methods outlined in [11]. Regression trees were fitted and optimised using five-fold valuation [9] and constructed using the “tree” library in the statistical software Splus (Version 6.2 Insightful Corp). Model accuracy was evaluated using 25% of the full dataset randomly selected prior to modelling. Models were assessed using receiver operator curves (ROC) and the area under the curve (AUC) and kappa metrics were used to calculate P-Fair. Models were discarded if they had an AUC value of less than 0.7 [12]. AUC calculations were used to calculate P-Fair threshold, which provides a balance between true presence and true absence for a range of different habitat prevalence [11]. P-Fair were used to threshold model probability of occurrences producing presence absence predicted habitat maps. The maps for the modelled habitat based on the towed video imagery have exactly the same extent and spatial resolution as the multibeam (2.5 meters squared). A total of 34 predicted habitat classes were generated, 32 describe the probability (0-1) of a given habitat types (e.g. reef/gorgonians/bryozoans). The remaining two variables are categorical and describe the general biota and general substrate, specifying each cell as one of 4 general biota and one of 4 general substrate classes.

**Image analysis of BRUVS**

BRUVS data were used to identify fish species, and estimate relative abundance and individual fork lengths. Relative abundance was defined as the maximum number of individuals of a given species present in the field of view of the right camera at the same time (‘MaxN’; [3,13]). This measure avoids repeated counts of the same individual and gives a conservative measure of relative abundance, as often only a portion of the total number of individuals in the area may be viewed at one time [14]. Following a strict calibration procedure (CAL; SeaGIS, 2008), measurements of fork length (snout to fork; FL) were then obtained using PhotoMeasure at time of MaxN [15]. Measurements of length were limited to those recorded within a maximum distance of 7 m from the cameras, which ensured accurate and precise measurements as well as a standardised sampling unit [16].

**Classification of functional group**

Herbivores were identified as plant eating species with trophic levels less than 2.5. Zooplanktivores were identified as species eating zooplankton in the water column and their trophic level varied from 2.7-3.7. Benthic omnivores and zoobenthivores were discriminated on the basis of their trophic levels with the former varying between 2.7 and 3.5 and the latter varying between 3.5 and 4.0. The former’s diet included a greater proportion of plant material with the latter having more invertebrates and small fish in their diet. Finally, piscivores were those species primarily eating nekton with their trophic levels varying between 3.6 and 4.5. The large number of benthic omnivores and benthic zoobenthivores and the large variation in their body size (and thus ecology) led us to separate them into small and large species within both groups. As a result small and large omnibenthivores were separated at a mean observed size of 31.5 cm while small and large zoobenthivores were separated at a mean observed size of 35.0 cm.

**Supplementary results**

**Study site**

The 250 km^2^ study location varied from 5 to 160 m depth and included a diversity of habitats. Abiotic habitats observed directly by cameras were comprised of high profile reef (HPR; 3.1%), medium profile reef (MPR; 25.2%), low profile reef (LPR; 20.9%), sand inundated reef (SIR; 23.3%), sand (SA; 23.5%) and undetermined (4.0%) while biotic habitats were comprised of kelp (KELP; 37.3%), other macroalgae (MAC; 12.6%), sessile invertebrates (SI; 12.3%), seagrass (SG; 0.6%), bare (BA, 33.2%) and undetermined (4.0%).

A total of 19,361 fish were identified from 139 species representing 57 families. Of these species, 66.2% were endemic to Australia and 49.6% were target species for either commercial or recreational fisheries. Species were moderately variable in vulnerability (mean = 44.1 (range 10-87); CV = 42.4%) but less variable in terms of trophic level (mean = 3.4 (range 2-4.5); CV = 18%). Small benthic omnivores made up the most abundant trophic category (24.5%) followed by nekton feeders (23.7%), small zoobenthivores (18.0%), large zoobenthivores (10.1%), herbivores (8.6%), zooplanktivores (7.9%) and large benthic omnivores (7.6%).

Estimated biodiversity metrics also varied by sample (Table 1, main text). Species richness was moderately variable, ranging from 1-31 species per sample (mean = 10.8) (Table 1). Abundance and biomass were more variable, reflecting the presence of small schooling species on some deployments or the presence of rare large individuals, respectively. The percentage of vulnerable species reflected this variability, with biomass-weighted estimates being higher than abundance, reflecting the presence of large relatively rare individuals. Target species tend to be larger than non-target species and many endemics are small. Thus the % target species was higher when weighted by biomass and % endemic species was higher when weighted by abundance.

**References**

1. Stevens DL, Olsen AR. Spatially Balanced Sampling of Natural Resources. J Am Stat Assoc. 2004;99: 262–278. doi:10.1198/016214504000000250

2. Roland Pitcher C, Lawton P, Ellis N, Smith SJ, Incze LS, Wei C-L, et al. Exploring the role of environmental variables in shaping patterns of seabed biodiversity composition in regional-scale ecosystems. J Appl Ecol. 2012;49: 670–679. doi:10.1111/j.1365-2664.2012.02148.x

3. Cappo M, Harvey E, Malcolm H, Speare P. Potential of video techniques to monitor diversity, abundance and size of fish in studies of Marine Protected Areas. pp. 455-464. Beumer JP, Grant A and Smith DC Aquatic Protected Areas - what works best and how do we know? World Congress on Aquatic Prote. 2003.

4. Watson D, Harvey E, Fitzpatrick B. Assessing reef fish assemblage structure: how do different stereo-video techniques compare? Mar Biol. 2010; 1–31. Available: http://link.springer.com/article/10.1007/s00227-010-1404-x

5. Watson DL, Harvey ES, Anderson MJ, Kendrick G a. A comparison of temperate reef fish assemblages recorded by three underwater stereo-video techniques. Mar Biol. 2005;148: 415–425. doi:10.1007/s00227-005-0090-6

6. Harvey E, Shortis M. A system for stereo-video measurement of sub-tidal organisms. Mar Technol Soc J. 1996;29: 10–22.

7. Harvey E, Fletcher D, Shortis M. Estimation of reef fish length by divers and by stereo-video: a first comparison of the accuracy and precision in the field on living fish under operational conditions. Fish Res. 2002;57: 255–265.

8. Stevens DL, Olsen AR, Althaus F, Hill N, Ferrari R, Edwards L, et al. A Standardised Vocabulary for Identifying Benthic Biota and Substrata from Underwater Imagery : The CATAMI Classification Scheme. 2003;610: 1–18. doi:10.1371/journal.pone.0141039

9. Breiman L, Friedman J, Stone CJ, Olshen RA. Classification and regression trees. CRC press. 1984.

10. De’ath G, Fabricius K. Classification and regression trees: a powerful yet simple technique for ecological data analysis. Ecology. 2000;81: 3178–3192.

11. Holmes KW, Van Niel KP, Radford B, Kendrick G a., Grove SL. Modelling distribution of marine benthos from hydroacoustics and underwater video. Cont Shelf Res. 2008;28: 1800–1810. doi:10.1016/j.csr.2008.04.016

12. Hosmer Jr DW, Lemeshow S, Sturdivant., X. R. Model‐building strategies and methods for logistic regression. In Applied Logistic Regression, third edition. Wiley. 2000.

13. Priede I., Bagley P, Smith A, Creasey S, Merretta NR. Scavenging deep demersal fishes of the Porcupine Seabight, north-east Atlantic: observations by baited camera, trap and trawl. J Mar Biol Assoc United Kingdom. 1994;74: 481 – 498.

14. Willis TJ, Millar RB, Babcock RC. Detection of spatial variability in relative density of fishes: comparison of visual census, angling, and baited underwater video. Mar Ecol Prog Ser. 2000;198: , 249–260.

15. SeaGIS. PhotoMeasure and EventMeasure. SeaGIS Pty Ltd. www.seagis.com.au. PhotoMeasure and EventMeasure. SeaGIS Pty Ltd. www.seagis.com.au. 2008.

16. Harvey E, Shortis M, Stadler M, Cappo M. A comparison of the accuracy and precision of measurements from single and stereo-video systems. Mar Technol Soc J. 2002;36: 38–49.
