# Supplementary material for: Models of Marine Fish Biodiversity: Assessing Predictors from Three Habitat Classification Schemes
Source: PLoS One. 2016 Jun 22;11(6):e0155634. doi: 10.1371/journal.pone.0155634 (PMC4917103; doi:10.1371/journal.pone.0155634)
Supplement: S1 Table — (DOCX) [file pone.0155634.s002.docx]

**S1 Table**. Description of all potential predictor variables

| **Data type** | **Variable** | **Code** | **Description** |
| --- | --- | --- | --- |
|  | Depth | DEPTH | Depth (m) estimated at the time of sampling |
| **Direct  observer habitat  classes** | Abiotic | HPR | High profile reef |
|  |  | MPR | Medium profile reef |
|  |  | LPR | Low profile reef |
|  |  | SIR | Sand inundated reef |
|  |  | SA | Sand |
|  | Biotic | COR | Coral |
|  |  | KELP | Kelp |
|  |  | MAC | Other macroalgae |
|  |  | SA | No biota |
|  |  | SG | Seagrass |
|  |  | SI | Noncoral sessile inverts |
| **Multibeam** | Aspect | asp | Azimuthal direction of the steepest slope, calculated on a 3 x 3 cell area |
|  | Bathymetry | bathy | Elevation relative to the Australian Height Datum (AHD) |
|  | Curvature | curv | Combined index of profile and plan curvature |
|  | Hypsometric index (12.5m radius) ^ | hyp5 | Indicator of whether a cell is a high or low point within the local neighbourhood |
|  | Hypsometric index (25 m radius) ^ | hyp10 |  |
|  | Hypsometric index (62.5 m radius) ^ | hyp25 |  |
|  | Hypsometric index (125 m radius) ^ | hyp50 |  |
|  | Morans I bathymetry (12.5 m radius) ^ | morb5 | A weighted correlation coefficient used to detect spatial dependence.  Calculated on the residuals from a linear trend surface. |
|  | Morans I bathymetry (25 m radius) ^ | morb10 |  |
|  | Morans I bathymetry (62.5 m radius) ^ | morb25 |  |
|  | Morans I bathymetry (125 m radius) ^ | morb50 |  |
|  | Plan Curvature | plan | Second derivative of elevation: concavity/convexity perpendicular to the slope, calculated on a 3 x 3 cell area |
|  | Profile Curvature | prof | Second derivative of elevation: concavity/convexity parallel to the slope, calculated on a 3 x 3 cell area |
|  | Trend | trend | The linear trend calculated across the bathymetry dataset |
|  | Depth Residuals | resid | Depth minus trend |
|  | Range (12.5 m radius) ^ | rng5 | Maximum minus the minimum elevation in a local neighbourhood (local relief) |
|  | Range (25 m radius) ^ | rng10 |  |
|  | Range (62.5 m radius) ^ | Rng25 |  |
|  | Range (125 m radius) ^ | rng50 |  |
|  | Slope | slp | First derivative of elevation: Average change in elevation / distance calculated on a 3 x 3 cell area |
|  | Snippits | snip | Second return from multibeam, indicator of texture, density |
|  | Standard Deviation (12.5 m radius) ^ | std5 | Standard deviation of elevation |
|  | Standard Deviation (25 m radius) ^ | std10 |  |
|  | Standard Deviation (62.5 m radius) ^ | Std25 |  |
|  | Standard Deviation (125 m radius) ^ | std50 |  |
|  | Rugosity (surface ratio) | srfratio | Surface area, calculated as a ratio of neighbouring cells (Jenness, 2002) |
|  | Rugosity (surface area) | srfarea | Surface area per pixel, calculated using the eight neighbouring cells (Jenness, 2002) |
| **Predicted  habitat  classes** | Kelp | kelp |  |
|  | Sessile Invertebrates | si |  |
|  | Other algae | othalg |  |
|  | Rhodoliths | rodo |  |
|  | Seagrass | sg |  |
|  | Vegetation | veg |  |
|  | General Substrate | gen_sub |  |
|  | Gravel | gravel |  |
|  | Reef - high relief | rfhigh |  |
|  | Reef – low relief | rflow |  |
|  | Reef - medium relief | rfmed |  |
|  | Reef | reef |  |
|  | Sand | sand |  |
|  | Obscured reef | obsrf |  |
|  | Sediment | sed |  |
|  | Sediment - fine | sedflt |  |
|  | Sediment medium | sedmed |  |
|  | Algae - mixed | algmix |  |
|  | Algae - red | algred |  |
|  | Algae -understory | algund |  |
|  | Seagrass - amphibolis | amphib |  |
|  | Macroalgae | macalg |  |
|  | Sponge | sponge |  |
|  | Seagrass - posidonia | posid |  |
|  | Sediments - fine | sedfin |  |
|  | Bryozoans | bryozo |  |
|  | Brown algae | balgae |  |
|  | Ascidians | ascidia |  |
|  | Scytophalia | scytot |  |
|  | Red algae | ralgae |  |
|  | Pyura | pyura |  |
|  | Hard coral | harcor |  |
|  | Gorgonians | gorgon |  |
| ^ Local neighborhood analysis. Original cell size is 2.5m | | | |
