# Supplementary material for: Models of Marine Fish Biodiversity: Assessing Predictors from Three Habitat Classification Schemes
Source: PLoS One. 2016 Jun 22;11(6):e0155634. doi: 10.1371/journal.pone.0155634 (PMC4917103; doi:10.1371/journal.pone.0155634)
Supplement: S2 Table — (DOCX) [file pone.0155634.s003.docx]

**S2 Table**. Parameters for ‘best’ boosted regression tree models for nine biodiversity metrics, developed with five sets of predictors. Predictors were developed from three sets of habitat data: multibeam habitats (multibeam), predicted habitats (pred. habitats) and direct observer habitats (biotic and abiotic categories). All models also contained depth as a predictor. Vulnerability, target and endemic metrics were developed for both the percentage of the total abundance and the percentage of the total biomass.

|  |  | **Predictors** | | | | |
| --- | --- | --- | --- | --- | --- | --- |
| **Metrics** | **Parameters** | Multibeam, depth,  biotic & abiotic | Pred.habitats, depth,  biotic & abiotic | Multibeam & depth | Pred. habitats & depth | Biotic, abiotic  & depth |
| Total species richness | Learning rate | 0.01 | 0.005 | 0.01 | 0.01 | 0.01 |
|  | Tree complexity | 2 | 3 | 3 | 3 | 3 |
| Total  abundance | Learning rate | 0.01 | 0.01 | 0.001 | 0.001 | 0.01 |
|  | Tree complexity | 3 | 3 | 3 | 3 | 3 |
| Total biomass | Learning rate | 0.001 | 0.01 | 0.001 | 0.010 | 0.001 |
|  | Tree complexity | 3 | 2 | 2 | 2 | 3 |
| Vulnerability (abundance) | Learning rate | 0.001 | 0.001 | 0.001 | 0.001 | 0.01 |
|  | Tree complexity | 3 | 2 | 3 | 3 | 3 |
| Vulnerability (biomass) | Learning rate | 0.001 | 0.001 | 0.001 | 0.01 | 0.001 |
|  | Tree complexity | 3 | 3 | 3 | 3 | 3 |
| Target (abundance) | Learning rate | 0.01 | 0.001 | 0.010 | 0.001 | 0.01 |
|  | Tree complexity | 2 | 3 | 3 | 2 | 3.000 |
| Target (biomass) | Learning rate | 0.001 | 0.001 | 0.010 | 0.001 | 0.001 |
|  | Tree complexity | 3 | 3 | 3 | 2 | 2 |
| Endemic (abundance) | Learning rate | 0.001 | 0.001 | 0.001 | 0.001 | 0.001 |
|  | Tree complexity | 2 | 2 | 3 | 3 | 3 |
| Endemic (biomass) | Learning rate | 0.001 | 0.001 | 0.001 | 0.001 | 0.001 |
|  | Tree complexity | 3 | 2 | 2 | 3 | 3 |
