# Supplementary material for: Models of Marine Fish Biodiversity: Assessing Predictors from Three Habitat Classification Schemes
Source: PLoS One. 2016 Jun 22;11(6):e0155634. doi: 10.1371/journal.pone.0155634 (PMC4917103; doi:10.1371/journal.pone.0155634)
Supplement: S3 Table — (DOCX) [file pone.0155634.s004.docx]

**S3 Table.** Ranges of CV Deviance and mean prediction error for five runs of the ‘best’ boosted regression tree models for nine biodiversity metrics, developed with five sets of predictors. Predictors were developed from three sets of habitat data: multibeam habitats (multibeam), predicted habitats (pred. habitats) and direct observer habitats (biotic and abiotic categories). All models also contained depth as a predictor. Vulnerability, target and endemic metrics were developed for both the percentage of the total abundance and the percentage of the total biomass.

|  |  | **Predictors** | | | | |
| --- | --- | --- | --- | --- | --- | --- |
|  | **Metrics** | Multibeam, depth,  biotic & abiotic | Pred.habitats, depth,  biotic & abiotic | Multibeam & depth | Pred.habitats & depth | Biotic, abiotic  & depth |
| **CV deviance  explained** | Species richness | 62.51 – 64.09 | 61.09 – 65.43 | 53.61 – 56.71 | 48.01 – 50.15 | 57.78 - 59.41 |
|  | Total abundance | 42.3 - 44.26 | 40.95 - 43.29 | 38.20 - 39.8 | 33.87 - 35.27 | 38.40 - 40.31 |
|  | Total biomass | 15.7 - 17.12 | 16.8 - 18.01 | 13.8 - 15.91 | 16.21 - 18.3 | 13.8 - 14.3 |
|  | Vulnerability (abundance) | 25.44 - 26.2 | 26.16 - 27.57 | 22.44 - 24.36 | 21.97 - 22.14 | 23.01 - 23.97 |
|  | Vulnerability (biomass) | 0.02 - 0.8 | 1.27 - 1.96 | 0.17 - 0.6 | 0.05 - 1.73 | 0.49 - 1.49 |
|  | Target (abundance) | 25.22 - 28.25 | 26.88 - 26.99 | 18.38 - 20.12 | 10.54 - 12.34 | 22.64 - 25.76 |
|  | Target (biomass) | 10.5 - 13.65 | 7.12 - 9.99 | 7.70 - 9.81 | 2.15 - 3.51 | 6.4 - 7.72 |
|  | Endemic (abundance) | 8.33 - 9.15 | 9.716 - 10.29 | 7.95 - 8.71 | 9.21 - 9.72 | 4.54 - 6.37 |
|  | Endemic (biomass) | 5.96 - 6.86 | 8.14 - 8.7 | 5.35 - 7.79 | 7.49 - 8.9 | 2.08 - 3.26 |
| **Mean prediction  error** | Species (richness) | 10.26 – 10.38 | 11.79 – 12.05 | 11.3 – 11.65 | 13.29 – 15.01 | 13.19 – 13.92 |
|  | Total (abundance) | 13.2 - 13.51 | 15.3 - 15.51 | 16.8 - 17.13 | 19.6 - 19.79 | 18.1 - 18.3 |
|  | Total (biomass) | 8.1 - 8.2 | 8.3 - 8.6 | 8.0 - 8.51 | 8.49 - 8.6 | 8.5 - 8.7 |
|  | Vulnerability (abundance) | 10.81 - 10.9 | 11.78 - 12.05 | 10.8 - 11.63 | 11.86 - 12.7 | 12.08 - 12.47 |
|  | Vulnerability (biomass) | 15.23 - 15.49 | 15.28 - 15.59 | 15.4 - 15.78 | 15.51 - 15.8 | 15.62 - 15.94 |
|  | Target (abundance) | 61.16 - 68.66 | 74.9 - 79.29 | 65.28 - 82.1 | 94.75 - 100.9 | 75.99 - 76.75 |
|  | Target (biomass) | 57.7 - 68.2 | 60.7 - 78.12 | 60.7 - 78.09 | 68.9 - 98.1 | 76.79 - 99.32 |
|  | Endemic (abundance) | 45.6 - 50.11 | 68.22 - 77.5 | 49.25 - 55.68 | 58.46 - 69.52 | 54.7 - 55.35 |
|  | Endemic (biomass) | 230.4 - 246.9 | 244.5 - 261.4 | 241.2 - 267.9 | 238.9 - 265.7 | 311.7 - 316.3 |
