# Supplementary material for: Models of Marine Fish Biodiversity: Assessing Predictors from Three Habitat Classification Schemes
Source: PLoS One. 2016 Jun 22;11(6):e0155634. doi: 10.1371/journal.pone.0155634 (PMC4917103; doi:10.1371/journal.pone.0155634)
Supplement: S4 Table — (DOCX) [file pone.0155634.s005.docx]

**S4 Table.** List of predictor variables selected for boosted regression tree model development. Variables are divided into three sets (plus depth) reflecting the three different types of habitat data used. The two variables of the direct observer habitat classification are categorical, with a total of 5 abiotic and six biotic categories.

| **Multibeam** | **Predicted habitats** | **Direct observer habitats** | **Other** |
| --- | --- | --- | --- |
| Range (12.5m) | Sediment | Abiotic | Depth |
| Slope | Vegetation type | Biotic |  |
| Hypsometric (125m) | Sessile inverts |  |  |
| Curvature | Sponge |  |  |
| Depth residuals | Rodoliths |  |  |
| Hypsometric (12.5m) | Reef |  |  |
| Aspect | Other algae |  |  |
| SD of elevation (12.5m) | General biota |  |  |
| Trend | General substrate |  |  |
| SD of elevation (125m) | Seagrass |  |  |
| Snippits | Bryozoan |  |  |
| Range (125m) |  |  |  |
